# Safety and efficacy of the rSh28GST urinary schistosomiasis vaccine: A phase 3 randomized, controlled trial in Senegalese children

**DOI:** 10.1371/journal.pntd.0006968

**Published:** 2018-12-07

**Authors:** Gilles Riveau, Anne-Marie Schacht, Jean-Pierre Dompnier, Dominique Deplanque, Modou Seck, Nawal Waucquier, Simon Senghor, Delphine Delcroix-Genete, Emmanuel Hermann, Noureddine Idris-Khodja, Claire Levy-Marchal, Monique Capron, André Capron

**Affiliations:** 1 Biomedical Research Center EPLS, Saint Louis, Senegal; 2 CIIL—Center for Infection and Immunity of Lille, Institut Pasteur de Lille, Univ. Lille, CNRS UMR, Inserm U1019—Lille, France; 3 Univ. Lille, Inserm, CHU Lille, CIC—Centre d’investigation clinique, Lille, France; 4 INSERM, UMR, Regenerative Nanomedicine (RNM), FMTS, Strasbourg, France; 5 Pole de Recherche Clinique Inserm, Paris, France; 6 Univ. Lille, Inserm, CHU Lille, LIRIC U 995, Lille Inflammation Research International Center Lille, France; 7 Académie des Sciences, 23 Quai de Conti, Paris, France; Queen's University Belfast, UNITED KINGDOM

## Abstract

**Background:**

Urinary schistosomiasis, the result of infection by Schistosoma haematobium (Sh), remains a major global health concern. A schistosome vaccine could represent a breakthrough in schistosomiasis control strategies, which are presently based on treatment with praziquantel (PZQ). We report the safety and efficacy of the vaccine candidate recombinant 28-kDa glutathione S-transferase of Sh (rSh28GST) designated as Bilhvax, in a phase 3 trial conducted in Senegal.

**Methods and findings:**

After clearance of their ongoing schistosomiasis infection with two doses of PZQ, 250 children aged 6–9 years were randomized to receive three subcutaneous injections of either rSh28GST/Alhydrogel (Bilhvax group) or Alhydrogel alone (control group) at week 0 (W0), W4, and W8 and then a booster at W52 (one year after the first injection). PZQ treatment was given at W44, according to previous phase 2 results. The primary endpoint of the analysis was efficacy, evaluated as a delay of recurrence of urinary schistosomiasis, defined by a microhematuria associated with at least one living Sh egg in urine from baseline to W152. During the 152-week follow-up period, there was no difference between study arms in the incidence of serious adverse events. The median follow-up time for subjects without recurrence was 22.9 months for the Bilhvax group and 18.8 months for the control group (log-rank p = 0.27). At W152, 108 children had experienced at least one recurrence in the Bilhvax group versus 112 in the control group. Specific immunoglobulin (Ig)G1, IgG2, and IgG4, but not IgG3 or IgA titers, were increased in the vaccine group.

**Conclusions:**

While Bilhvax was immunogenic and well tolerated by infected children, a sufficient efficacy was not reached. The lack of effect may be the result of several factors, including interference by individual PZQ treatments administered each time a child was found infected, or the chosen vaccine-injection regimen favoring blocking IgG4 rather than protective IgG3 antibodies. These observations contrasting with results obtained in experimental models will help in the design of future trials.

**Trial registration:**

ClinicalTrials.gov NCT 00870649

## Author summary

Vaccines represent an attractive tool in the fight against schistosomiasis. Pre-clinical immunization studies with the schistosome enzyme 28 kDa glutathione *S*-transferase (28GST) have shown a significant reduction of schistosome egg production and subsequent pathology. The objective of the present phase 3 trial was to assess the efficacy and safety of vaccination with the recombinant 28GST of *Schistosoma haematobium* (rSh28GST) named Bilhvax, in infected school children. After Praziquantel treatment before inclusion and three administrations of rSh28GST at one month interval as primo-vaccination, subjects received a boost injection one year after the first administration. The efficacy was evaluated as a delay of recurrence of urinary schistosomiasis. While immunological analysis showed that Bilhvax induced a consistent immune response characterized by antibodies able to inhibit 28GST enzymatic activity, the efficacy endpoint was not reached. This lack of significant effect may be due to the negative conjunction of a too challenging recurrence criterion associated with safety measures ensuring repeated PZQ treatment. The control of these main factors will be essential for the subsequent trials and must provide evidence of the Bilhvax efficacy as a safe vaccine against uro-genital schistosomiasis.

## Introduction

Schistosomiasis is a chronic parasitic disease caused by trematodes that lay eggs in the urinary or gastrointestinal tract blood vessels [1]. It is associated with gastrointestinal or genitourinary disorders, pain, anemia, malnutrition, fatigue, and reduced exercise tolerance. These effects imply a loss of performance in parasitized individuals, especially schoolchildren, that hampers personal and community development [2]. In addition to the possible lethal outcome of the infection, the physical disability and social discomfort caused by schistosomiasis are tremendous, and meta-analyses have estimated that the current disease burden may exceed 70 million disability-adjusted life years [3].

Although 260 million people are infected by different *Schistosoma* species, and more than 200,000 deaths per year are registered, schistosomiasis remains a neglected disease [4]. Fewer than 40 million of those infected have received the unique drug available, praziquantel (PZQ), which has several limitations, including a lack of effect on reinfection and increased risk for emergence of drug-resistant parasites. This absence of a long-term efficient treatment emphasizes the need to develop a safe and efficacious vaccine that can be integrated into the control strategies for reducing schistosomiasis transmission and reinfection [5].

Closely associated with the parasite metabolism, the 28-kDa glutathione S-transferases (P28GSTs) have been identified in schistosomes as potent modulators of epithelial Langerhans and dendritic cell migration during infection [6], hormonal carriers for schistosomes [7], and the main enzymes involved in detoxification and antioxidant pathways [8]. Schistosome P28GSTs are potential vaccine candidates and have been extensively studied in various experimental models [1]. In addition to P28GST from *Schistosoma mansoni* (*Sm*) Sm28GST, P28GST from *Schistosoma haematobium* (*Sh*) Sh28GST has been further characterized, from molecular cloning to crystallization [9], and developed as a schistosome vaccine in non-human primates [10]. Indeed, Sh28GST can significantly reduce *Sh* worm fecundity in experimentally infected primates [10]. In addition, the combination of PZQ chemotherapy with 28GST DNA vaccination has been assessed in the mouse, triggering an enhanced specific immune response and decreased schistosomiasis pathology [11]. A phase 1 clinical trial conducted in healthy subjects demonstrated that the recombinant Sh28GST (rSh28GST) adsorbed to Alhydrogel did not induce significant toxicity in healthy adults and generated a Th2-type immune response characterized by cytokine and antibody profiles [12]. Phase 2 clinical testing showed that Bilhvax in combination with PZQ treatment was safe for infected adults and children (the Bilhvax program) [13] (Riveau et al, in preparation). More than 80% of the vaccinees included in these phases (adults 18–30 years, children 6–9 years) had a specific immune response following two administrations of Bilhvax at one-month intervals.

Here, we describe the results of a phase 3 trial of rSh28GST adjuvanted with Alhydrogel (Bilhvax). This phase 3 trial was designed to investigate the safety, efficacy, and immunogenicity of Bilhvax administered to infected Senegalese schoolchildren. In this clinical trial, Senegalese *Sh*-infected children aged 6 to 9 years were first cleared of their current schistosomiasis infection by a double PZQ treatment before receiving three injections of Bilhvax at one-month intervals, and then PZQ treatment before a booster one year after the first injection. Immediate and delayed tolerance as well as efficacy and immunogenicity of Bilhvax were studied.

The main objective of this randomized controlled trial was to show that co-administration of Bilhvax with PZQ could delay the risk of *Sh* clinical recurrence during the 3 years following vaccine administration to *Sh-*infected children living in an endemic area. The safety was monitored in these infected children according to clinical evidence, whereas immune response was followed in both Bilhvax and control groups by determining specific antibody titers as well as neutralizing antibodies.

## Methods

### Ethics statement

The trial protocol was approved and tial twice audited by the Senegalese Ethical Committee (Comité National d’Ethique de la Recherche en Santé; CNERS, Dakar, Senegal; Registration number SEN 14/08). The study was conducted in accordance with the Declaration of Helsinki III and with the International Ethical Guidelines for Biomedical Research Involving Human Subjects, as laid down by the Council for International Organizations of Medical Sciences in collaboration with the World Health Organization and the Good Clinical Practice guideline CPMP/ICH/135/95 [14–16]. Written informed consent was obtained from all parents or guardians prior to enrolment.

The trial was overseen by Inserm (Institut National de la Santé et de la Recherche Médicale, France) et WHO (NTD Department). This study is registered with ClinicalTrials.gov, number NCT 00870649

### Study design and participants

This randomized, parallel-group, controlled, double-blind phase 3 trial of the schistosome vaccine candidate Sh28GST (Bilhvax) was conducted at the Biomedical Research Center EPLS (Espoir Pour La Santé) and included 250 children living in 13 villages of the Saint-Louis region located in the lower Senegal River basin. The regional prevalence of urinary schistosomiasis in schoolchildren is estimated at over 60%. The school selection took place in two areas of the river valley, the Lampsar area (zone 1, from Saint Louis to Ross-Béthio) and the Djoudj area (zone 2, from Ross-Béthio to Ronkh). Zone 1 included 20 villages representing a total of 23 public schools and 20 Koranic schools (8237 children aged 5 to 14). Zone 2 included 10 villages with 10 public schools and 5 Koranic schools (2941 children). Mass treatment with PZQ and albendazole was performed in these 60 schools by the program, representing 11,178 children aged 5 to 14 treated before the start of the clinical trial.

Only Zone 1 was used for selection. In this area, 19 schools were selected based on the following criteria: presence of a school; agreement of the village chief; number of children >40 in the youngest classes (class CI and CP); more than 5 children with hematuria; village accessible during the rainy season; teacher collaboration; and no medical research intervention planned in the village. Among these 19 villages, 13 villages were selected, including a population of 2150 children corresponding to the age criterion. This population had a *Sh* prevalence of 44% with 692 children with hematuria >2+ (32%), and with egg load >50 eggs/10mL (16%). A total of 298 children were assessed for eligibility.

The trial was designed to evaluate the safety, immunogenicity, and efficacy of Bilhvax for prevention of clinical and parasitological recurrences of *Sh* infection during a 152-week follow-up period after the first injection in a population of schoolchildren aged 6–9 years. The primary efficacy endpoint was time to first recurrence of pathology due to *Sh* infection, anticipating a significant delay of first recurrence between vaccine and control groups during the 3-year period (W0/V1 to W152/V11). Secondary outcome measures were safety and immune response evaluation.

Written informed consent was obtained 8 W prior to randomization (W-8) from the children’s parents or guardians. The inclusion and exclusion criteria for participation in the trial are listed in Table 1.

**Table 1:** Inclusion and exclusion criteria for enrollment.

**Inclusion criteria**
- Male and female schoolchildren aged 6–9 years in the Saint-Louis region of Senegal
- Written informed consent obtained from the parents or guardians prior to enrolment.
- Subject in good basic health, based on medical history and physical examination
- Heavily infected with *S*. *haematobium* (≤50 *Sh* eggs per 10 ml urine found by urine filtration AND positive hematuria ≥ 2 +)
- Absence of severe urinary tract injuries detected by ultrasound examination
**Exclusion criteria**
- Child or parent participation refusal.
- Vaccination in the 3 months prior to enrolment or intention to immunize with any other vaccine(s) within 3 years after enrolment.
- Use of systemic corticosteroids within the 2 weeks prior to enrolment.
- Current administration of topical or nasal corticosteroids.
- Immunosuppressive therapy within the 4 weeks prior to enrolment.
- History of allergy or hypersensitivity to vaccines.
- Life-threatening illnesses in the short or medium term.
- Subjects who the investigator believes that their health state is not compatible with the requirement of the protocol.

**Fig 1:**
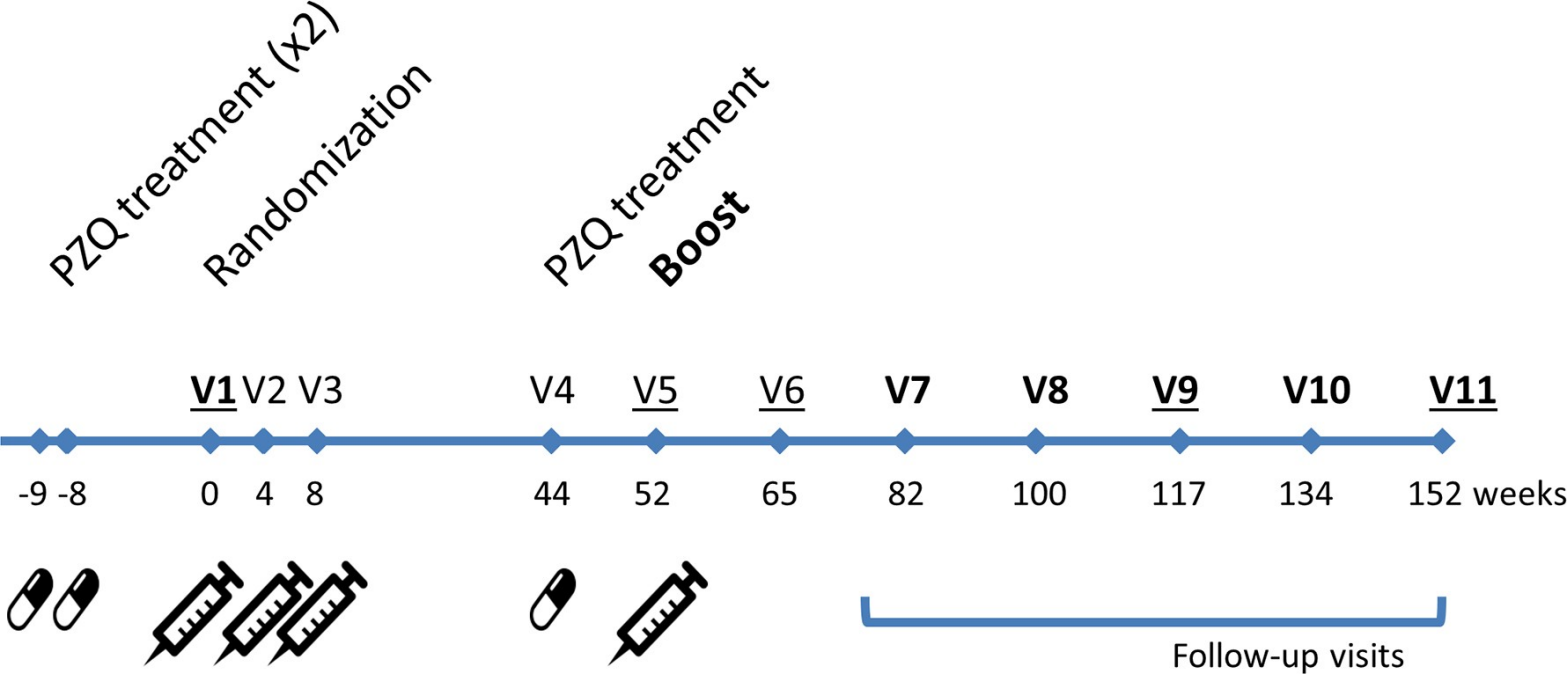
Timeline for vaccination, follow-up, and blood sampling schedules. Vx: Blood sampling; bold Vx: parasitological tests.

Each included participant was seen at 13 visits: 2 visits of pre-inclusion (Vp), at 9 weeks (W-9) and 8 weeks (W-8) prior randomization (respectively Vp1 and Vp2), the inclusion visit (W0), and 10 visits from W4 (visit V2) to W152 (visit V11) at 4, 8, 44, 52, 65, 82, 100, 117, 134, and 152 weeks after inclusion (Fig 1).

### Randomization and vaccination

From the pediatric population living in 13 villages of the urinary schistosomiasis hyperendemic region of the Lower Senegal River Valley, 298 children were preselected according to the two criteria for *Sh* infection: medium or high microhematuria (≥2+) and a minimum load of 50 eggs/10 ml of urine. Microhematuria was evaluated using a strip reader (Dipstick Multistix 8 SG, Bayer, Siemens/reader Clinitek Status) as “negative,” “trace,” “1+” (corresponding to 25 red blood cells (RBCs)/mm^3^), “2+” (corresponding to 80 RBC/mm^3^), and “3+” (corresponding to 200 RBC/mm^3^). A positive microhematuria was defined as urine coded ≥1+. Urine samples were collected in the morning over the course of 2 hours.

To clear their ongoing schistosomiasis infection, preselected children were treated twice with PZQ (40 mg/kg) at W-9/Vp1 and W-8/Vp2 prior to randomization. Participants were assigned randomly at W0/V1 in a 1:1 ratio to control or vaccine group. The “Bilhvax group” received three subcutaneous injections of rSh28GST formulated with Alhydrogel as adjuvant at W0/V1, W4/V2, and W8/V3, followed by a vaccine boost at W52/V5. The control group received four subcutaneous injections of Alhydrogel at W0/V1, W4/V2, W8/V3, and W52/V5. Both groups received one dose of 40 mg/kg PZQ at W44/V4, i.e., 8 weeks before the booster injection (Fig 1).

### Vaccine

Batches of rSh28GST were produced and purified from recombinant *Saccharomyces cerevisiae* culture (TGY73.4—pTG8889 strain) under Good Manufacturing Practice (GMP) conditions by Eurogentec S.A. (Belgium). The rSh28GST clinical batch (M-BIX-P01/189a) was lyophilized under GMP conditions by Miltenyi (Germany) at 253 μg (±10%) per vial. The lyophilized preparation was re-suspended extemporaneously using 1 ml of apyrogenic and sterile alum solution 0.2% (Al_2_O_3_ 0.2%; Al(OH)_3_ 3%; NaCl 9 g/L; ammonium carbonate buffer 10 mM, pH7.8) (Alhydrogel from Superfos, Denmark; batch **#**14093) and administered in a volume of 0.4 ml.

### Safety assessments

Adverse events (AEs), signs, and symptoms were defined and classified according to the DMID Safety Reporting and Pharmacovigilance for pediatric toxicity [17]. For general adverse effects, the prevalence and intensity of the following signs and symptoms were assessed: abdominal pain, vomiting, nausea, diarrhea, headache, sleepiness, fever, vertigo, and pruritus. Occurrence of local AEs at the injection site (pain, pruritus, or swelling) or any regional adverse reaction to vaccine or placebo injections was recorded 1 h after each vaccination during visits V1, V2, V3, V4 (PZQ treatment), and V5. Medical exams were also performed 4 h and 24 h after vaccinations during visits V3, V4, and V5. A questionnaire was given to parents to report whether their children felt any AE or needed any medical assistance at 48 h and 72 h after V1, V2, V3, V4, and V5. Parents of included children could be in contact (7/7; 24/24) with the medical team for any illnesses anytime over the course of the trial.

### Assessment of recurrence

The recurrence of *Sh* infection was defined as a positive microhematuria (≥1+) accompanied by the presence of at least one living egg in 10 ml of urine. Hematuria was tested during the follow-up visits (V7, V8, V9, V10, and V11) or following spontaneous complaint of the subject during inter-visit periods.

Positive hematuria (≥1+) was considered as not due to schistosomiasis when three consecutive urine filtrations (UFs) performed on three different days during one week were negative. When hematuria was established using the urinary strip, UFs were systematically performed with the urine collected. When UF was positive, the hatching assay was performed with the eggs contained in the remaining urine sample to evaluate their viability (expressed in % hatching).

At any time, if an individual complaint suggested urinary disorders, clinical examinations as well as parasitological tests were immediately performed. When *Sh* recurrence was established in a participant, an evaluation of *Sm* in stool sample using the Kato-Katz assay (KK) was performed [18, 19] because concomitant *Sh* and *Sm* infection is endemic in the selected villages.

### Laboratory procedures

Two-hour urine samples were collected during the morning at the selection (before inclusion) and then every 4 months during the scheduled follow-up visits (from W82/V7 to W152/V11). Parasitological evaluation was carried out when the urine sample was positive for hematuria (≥1+). Two samples of 10 ml each were separately filtrated and filters were analyzed by two different readers as described elsewhere [20]. In case of high discrepancy between the two counts, both filters were examined by a third reader, and if necessary, the filtration was repeated. At the last visit (the closing visit) (W152/V11), UF and KK were performed for each individual.

The hatching assay was carried in duplicate (2 wells on two separate 12-well plates) with the number of eggs close to 100 eggs per well. If the number of eggs collected proved insufficient, a single reaction well was completed and read by two different technicians. If the number of eggs counted in the reaction wells was less than 50, the percentage of hatching was not calculated, and only the presence or absence of hatched eggs was noted [21].

When *Sm* eggs were observed in urine (UF) and the KK test was negative, two consecutive stool samples were analyzed.

### Assessment of efficacy

The aim of the vaccine efficacy was to reduce the risk of *S*. *haematobium* pathology recurrences over the course of 3 years following vaccine administration in children exposed to urinary schistosomiasis. The primary endpoint of efficacy was time to first recurrence of pathology due to *Sh* infection, anticipating a significant delay of first recurrence between vaccine and control groups during the 3-year period from D0 (V1) to W152 (V11) (intention-to-treat (ITT) population) between the vaccine and control groups. The difference in delay to the onset of recurrence between both groups was also evaluated as the modified ITT (mITT) population considering the period post vaccination, V6 to V11.

Secondary efficacy endpoints were defined as the percentage of participants without recurrence, the number of recurrences per subject, and parasitological manifestations at the first recurrence including the number of viable eggs, the number of hatched eggs, and percent of eggs hatching.

Individuals were systematically treated with PZQ each time schistosomiasis infection/recurrence was identified.

### Assessment of immunogenicity

Specific anti-rSh28GST antibodies were measured by ELISA in individual sera at W0/V1 (background), W44/V4 (after three immunizations), W52/V5 (after PZQ treatment), W65/V6 (after booster), W117/V9, and W152/V11 according to Riveau et al [12]. Results are expressed as antibody titers. Titers were defined as the highest dilution yielding an absorbance two or three times above the negative control depending on the studied isotype. (wells containing the reference control negative pool of Senegalese sera instead of participant sera in the same plate). Individuals were considered as positive responders when the antibody titer was greater than the threshold defined as 3-fold the standard deviation above the mean titer value of all individuals at W0. Specific antigen (rSh28GST 10 μg/ml) was coated on 96-well plates (Nunc-immuno plate, F96 cert., Maxisorp, Roskilde Denmark) for 2.5 h at 37°C. After blocking with phosphate-buffered saline containing 0.5% gelatin (Merck, Darmstadt, Germany), serial dilutions of individual sera were added, and plates were incubated overnight at 4°C. Specific biotinylated monoclonal antibodies to human immunoglobulin (Ig) isotypes (BD Pharmingen G(h+l), G2, G4, A1A2, and E; Sigma G3; SB G1) were added (1.5 h at 37°C) at a 1/2000 dilution for total IgG; 1/4000 for IgG1 and IgG3; 1/3000 for IgG2, IgG4, and IgA1/A2; and 1/500 for IgE detection. IgM were not measured due to the lack of adequate reagent and cross reactivity with other immunoglobulin. Peroxidase-conjugated streptavidin (1/20000; 30 min at 37°C) was then added (SPA-BIOSPA, Milano). Colorimetric development was performed with ABTS [Sigma, liquid substrate 2.2’-azino-bis (3-ethylbenz-thiazoline-6-sulfonic acid)], and absorbance (OD) was measured at 405 nm (Reader BioTek- EL808). At each step of the ELISA procedure, the plate washing was performed using the plate washer (BioTek ELx405). Titers were defined as the highest dilution yielding an absorbance two or three times above the negative control depending on the studied isotype.

In addition to the titration of specific anti-rSh28GST antibodies, functional aspects of these antibodies were investigated by following inhibition of rSh28GST enzymatic activity by sera from each individual. The glutathione S-transferase (GST) neutralizing capacity of antibodies was evaluated as previously described [12]. Briefly, 20 μl of rSh28GST solution (4 μg/ml in 50 mM potassium phosphate at pH 6.5; corresponding to 2.85 picomole/reaction well) was incubated with 20 μl of human serum for 1 h at 37°C in Immulon 3 Plates (Nunc, Roskilde, Denmark). The enzymatic reaction was carried out using 1-chloro-2, 4, dinitrobenzene (Sigma, St. Louis, MO) substrate. Enzymatic reaction intensity was measured by OD at 340 nm at 37°C (every 15” during 3’) (BioTek EL808, softwareGEN5), and appropriate controls (enzyme without serum and tested serum alone) were added. The percentage of inhibition was calculated as the ratio of GST activity after serum incubation to the GST activity in the control. An inhibition ≥10% was considered significant. Because in epidemiological surveys, individual sera inhibiting the 28GST up to 60% are associated with a low level of egg output [22], we considered this threshold of inhibition as a criterion of efficacy for the antibody response.

### Ultrasonography

The pathology associated with *Sh* infection was assessed by image analysis of lesions in the urinary tract detected by ultrasonography using the Niamey score, defined by WHO [23]. Ultrasonography was performed at W-8/Vp2, W0/V1, W65/V6, W82/V7, W100/V8, W117/V9, W134/V10, and W152/V11.

### Statistical analysis

All selected participants living in this hyperendemic region for urinary schistosomiasis had *Sh* infection before inclusion and had a probability for reinfection over the course of the trial estimated close to 100% in the control group. With this consideration, it was estimated that the trial would have 80% power to show the effectiveness of the vaccine as compared with control with at least 103 subjects per arm. Because a 10% dropout rate was considered probable within the 36 months after the first vaccination trial, we specified 250 (125 per arm) children as the recruitment target for this study. The ITT population included all participants who received at least one dose of vaccine or control.

Numerical factors of the study were compared using a Student’s *t*-test. Categorical factors such as incidences of AEs were compared using the chi-square or Fisher’s exact test. Cumulative recurrence of schistosomiasis hematuria for 36 months after inclusion (ITT) or 24 months after the booster (PP) was estimated by the Kaplan–Meier method to evaluate vaccine efficacy, and the log-rank analysis was used to assess differences between the two groups.

Univariate and multivariate Cox regression analyses of time to events were carried out to test the treatment effect. The Statistical Analysis Software version 9.1 (SAS Cary, NC) was used for all statistical analyses, and statistical significance was defined as a two-tailed p<0.05.

## Results

### Study population

**Fig 2:**
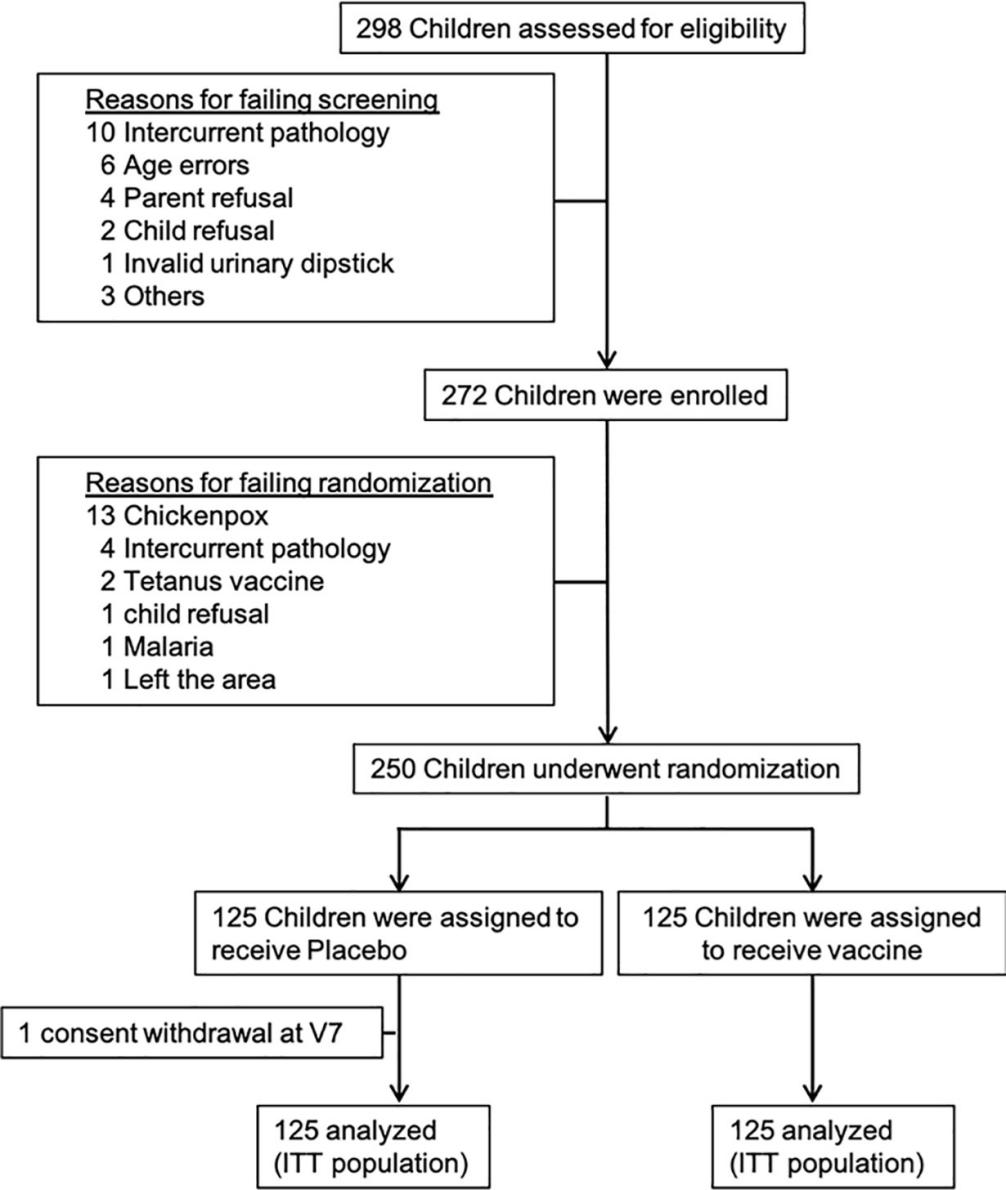
CONSORT diagram of children aged 6–9 years at enrollment and followed during 3 years after the first injection of Bilhvax or placebo. All enrolled children have received three doses of study vaccine or control, and were included in the ITT analysis.

Of the 298 schoolchildren assessed for eligibility, 272 subjects were enrolled, and 250 were included in the study in a short period of 25 days (Fig 2). The reasons for excluding subjects are presented in Fig 2 and Table 1. A total of 250 children (125 children in the vaccine group and 125 in the control group) were randomly assigned to receive either Bilhvax containing 100±10μg rSh28GST adjuvanted with Alhydrogel or 0.4 ml Alhydrogel as placebo in the control group. Arms were similar with respect to age, height, and weight at the time of enrollment (Table 2). The median duration of follow-up was 2.9±0.1 years, with no significant difference between the two groups. All included children received all planned doses of vaccine or placebo and PZQ treatment according to the study protocol and were included in the ITT protocol analysis. The primary endpoint was also analyzed with a modified ITT analysis, which was prospectively defined to exclude all recurrences observed before the boost.

**Table 2:** Baseline characteristics of population at V1.

*Groups*		*Control (n = 125)*	*Vaccine (n = 125)*	*Total (n = 250)*
**Age**	**6 years**	**26** (21%)	**25** (20%)	51 / 250 (20%)
	**7 years**	**50** (40%)	**49** (39%)	99 / 250 (40%)
	**8 years**	**34** (27%)	**37** (30%)	71 / 250 (28%)
	**9 years**	**15** (12%)	**14** (11%)	29 / 250 (12%)
	***Mean (std)***	***7*.*3*** *(0*.*9)*	***7*.*3*** *(0*.*9)*	*7*.*3 (0*.*9)*
**Gender**	**Boy**	**81** (65%)	**68** (54%)	149 / 250 (60%)
	**Girl**	**44** (35%)	**57** (46%)	101 / 250 (40%)
**Height (cm)**	**Min-Max**	110 to 141	112 to 143	110 to 143
	**Mean (std)**	**125** (7)	**125** (7)	125 (7)
	**Median [IQR]**	124 [120 ; 130]	124 [120 ; 131]	124 [120 ; 131]
**Weight (kg)**	**Min-Max**	15.9 to 29.0	16.7 to 31.4	15.9 to 31.4
	**Mean (std)**	**22.4** (3.0)	**22.3** (3.0)	22.4 (3.0)
	**Median [IQR]**	22.2 [20.1 ; 24.3]	22.2 [19.9 ; 24.4]	22.2 [20.0 ; 24.3]
**BMI**	**Min-Max**	12 to 18	12.2 to 16.8	12 to 18
	**Mean (std)**	**14.3** (1.0)	**14.2** (1.0)	14.3 (1.0)
	**Median [IQR]**	14.3 [13.5 ; 14.9]	14.1 [13.5 ; 14.9]	14.2 [13.5 ; 14.9]
**Hematuria (urine dipstick)**	**2+**	**23** (18%)	**23** (18%)	46 / 250 (18%)
	**3+**	**102** (82%)	**102** (82%)	204 / 250 (82%)
**Urinary filtration**	**Min-Max**	54 to 200	50 to 200	50 to 200
**Number of eggs/10 ml of urine**	**Mean (std)**	**133.9** (55.7)	**141.6** (60.5)	137.7 (58.1)
	**Median [IQR]**	124 [82 ; 200]	165 [79 ; 200]	135 [80. ; 200]

### Baseline characteristics

As summarized in Table 2, of 250 school children included in the study, 149 (59.6%) were boys and 101 (40.4%) were girls. The ages ranged from 6 to 9 years, with an average age of 7.3±0.9 years. The number in each age group was 51, 99, 71, and 29 for ages 6, 7, 8, and 9 years, respectively. Weight varied from 15.9 kg to 31.4 kg (average: 22.4±3.0 kg). The mean egg count among egg-excreting children was 137.7±58.1 eggs/10 ml of urine. Mean egg counts were 133.9±55.7 and 141.6±60.5 in the control and Bilhvax groups, respectively. Among 250 infected children included in the study, 97 (39%) excreted more than 200 eggs/10 ml of urine (41/125 in control group and 56/125 in the Bilhvax group). In terms of urinary tract morbidity due to *Sh* infection detected by ultrasonography, the score measured at Vp2 was 7.4 (range: 0–23) and 7.1 (range: 1–25) in the control and Bilhvax groups, respectively, with no significant differences between the two groups. Thus, variables such as age, gender, BMI, infection intensity at inclusion, severity of the pathology assessed by ultrasound on inclusion, were distributed in a uniform way within of both groups (control vs vaccinated). Statistical analysis found no relationship between these variables and the achievement of the primary endpoint of the study, nor with other endpoints such as immune response to vaccine or parasitological criteria such as egg viability.

### Safety

Among the 250 children, 247 (99%) (control, 123; Bilhvax, 124) presented a total of 1520 adverse events (control, 733; Bilhvax, 787) with a median of six adverse events per child (range: 3–8) during the 3-year study (2 AE/year/child). AEs of grade 1 represented 47.2% (n = 718) of the total AEs with 372 for the control group versus 346 for the vaccine group. Most frequent AEs were related to infectious diseases including schistosomiasis. Only 6% of the AEs were ≥grade 3 (control, 22; Bilhvax, 64), most of them local AEs at the injection site (Table 3).

**Table 3:** Main adverse events observed over the course of the study.

Type of adverse event	Control (n = 733)	Vaccine (n = 787)	Total (n = 1520)
**Local or general symptoms**	**88**	**145**	**233**
**Grade (1, 2, 3, 4)**	**(48,32,8,0)**	**(52,39,54,0)**	**(100, 71, 62, 0)**
Injection site induration	34	85	119
Pain at injection site	41	35	76
Pruritus at injection site	0	6	6
Fever	8	14	22
Influenza like syndrome	3	4	7
Other miscellaneous	2	1	3
**Infectious diseases**	**316**	**328**	**644**
**Grade (1, 2, 3, 4)**	**(163, 146, 7, 0)**	**(151, 170, 6, 1)**	**(314, 316, 13, 1)**
Schistosomiasis	131	128	259
Bronchitis	38	28	66
Gastroenteritis	21	21	42
Wound infection	16	21	37
Pyoderma	12	14	26
Tooth infection	12	14	26
Chicken pox	11	7	18
Other miscellaneous	75	109	184
**Gastrointestinal disorders**	**123**	**120**	**243**
**Grade (1, 2, 3, 4)**	**(82, 41, 0, 0)**	**(76, 44, 0, 0)**	**(158, 85, 0, 0)**
Abdominal pain	88	82	170
Vomiting or nausea	16	19	35
Diarrhea	11	13	24
Other miscellaneous	8	6	14
**Nervous system disorders**	**34**	**30**	**64**
**Grade (1, 2, 3, 4)**	**(26, 7, 1, 0)**	**(14, 16, 0, 0)**	**(40, 23, 1, 0)**
Headache	28	23	51
Sleepiness	4	6	10
Dizziness	1	0	1
Epilepsy	1	1	2
**Renal and urinary disorders**	**26**	**30**	**56**
**Grade (1, 2, 3, 4)**	**(23, 3, 0, 0)**	**(28, 2, 0, 0)**	**(51, 5, 0, 0)**
Hematuria	26	25	51
Other miscellaneous	0	5	5
**Surgical or medical interventions**	**53**	**54**	**107**
**Grade (1, 2, 3, 4)**	**(4, 49, 0, 0)**	**(0, 54, 0, 0)**	**(4, 103, 0, 0)**
Dental care	49	54	103
Surgery for inguinal hernia	2	0	2
Surgery for umbilical hernia	1	0	1
Dermabrasion	1	0	1
**All other adverse events**	**93**	**80**	**173**
**Grade (1, 2, 3, 4)**	**(26, 61, 6, 0)**	**(25, 52, 3, 0)**	**(51, 113, 9, 0)**

Data are presented as number of events and number for each grade level. Grades are classified as follow: mild (1); moderate (2); severe (3); potentially life-threatening complication (4)

All AEs possibly related to the treatment were identified in 74% children from the Bilhvax group and 58% children from the control group (p = 0.008). A total of 152 children (50% of the placebo group vs 72% in the vaccine group) experienced 418 local or regional adverse reactions after injection. In this context, the most frequent adverse reactions included both inflammation and pain at the site of injection. These local adverse reactions were more common in the vaccine group than in the control group (p<0.0001) (S1 Table). Nine children (six in control and three in the vaccine group, respectively) developed a total of 10 serious AEs (S2 Table), but none were life threatening or related to Bilhvax, and all were completely resolved.

### Vaccine efficacy

**Fig 3:**
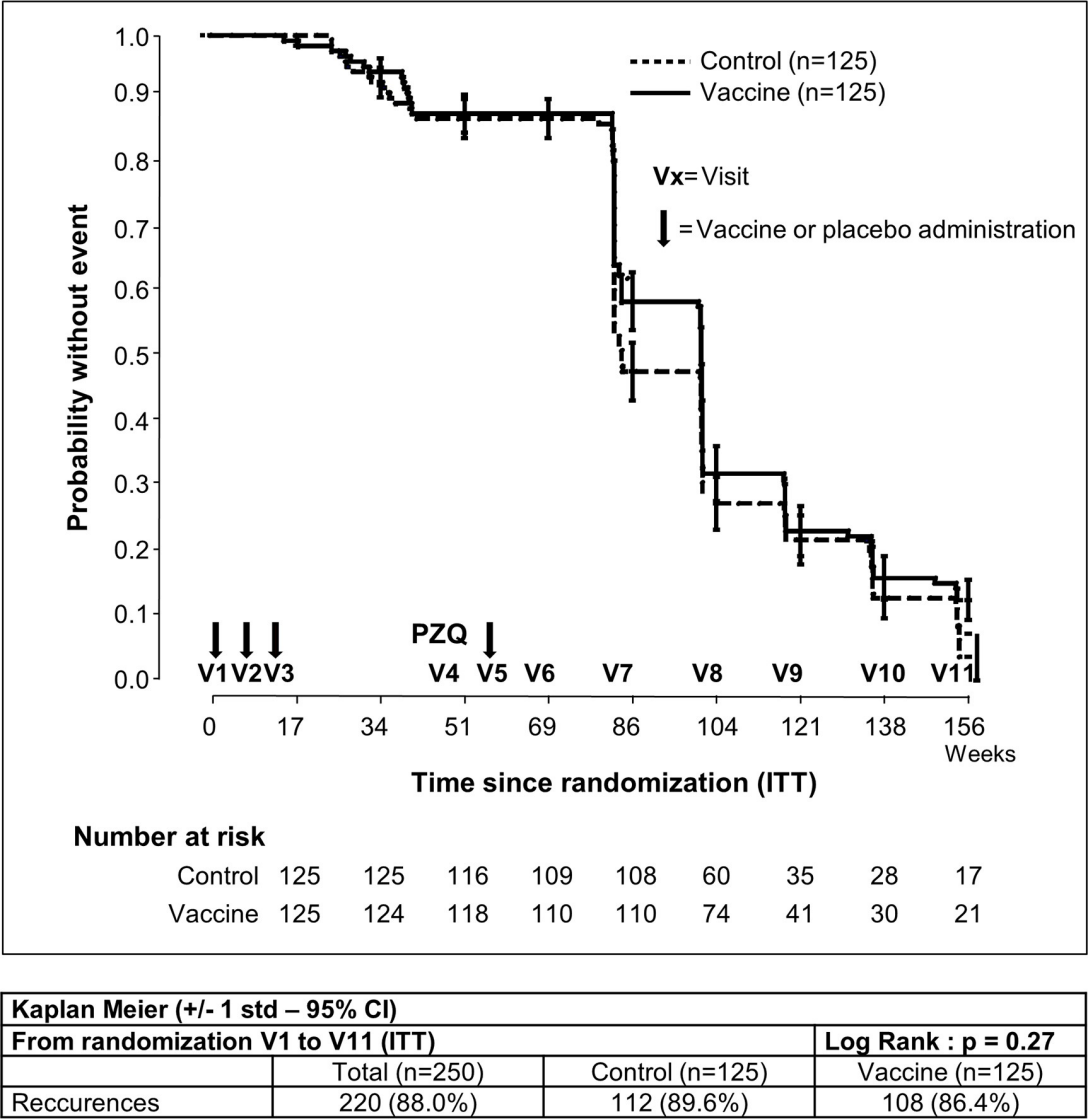
Kaplan–Meier estimates of the cumulative probability of not developing schistosomiasis recurrences among participants in the ITT analysis.

**Fig 4:**
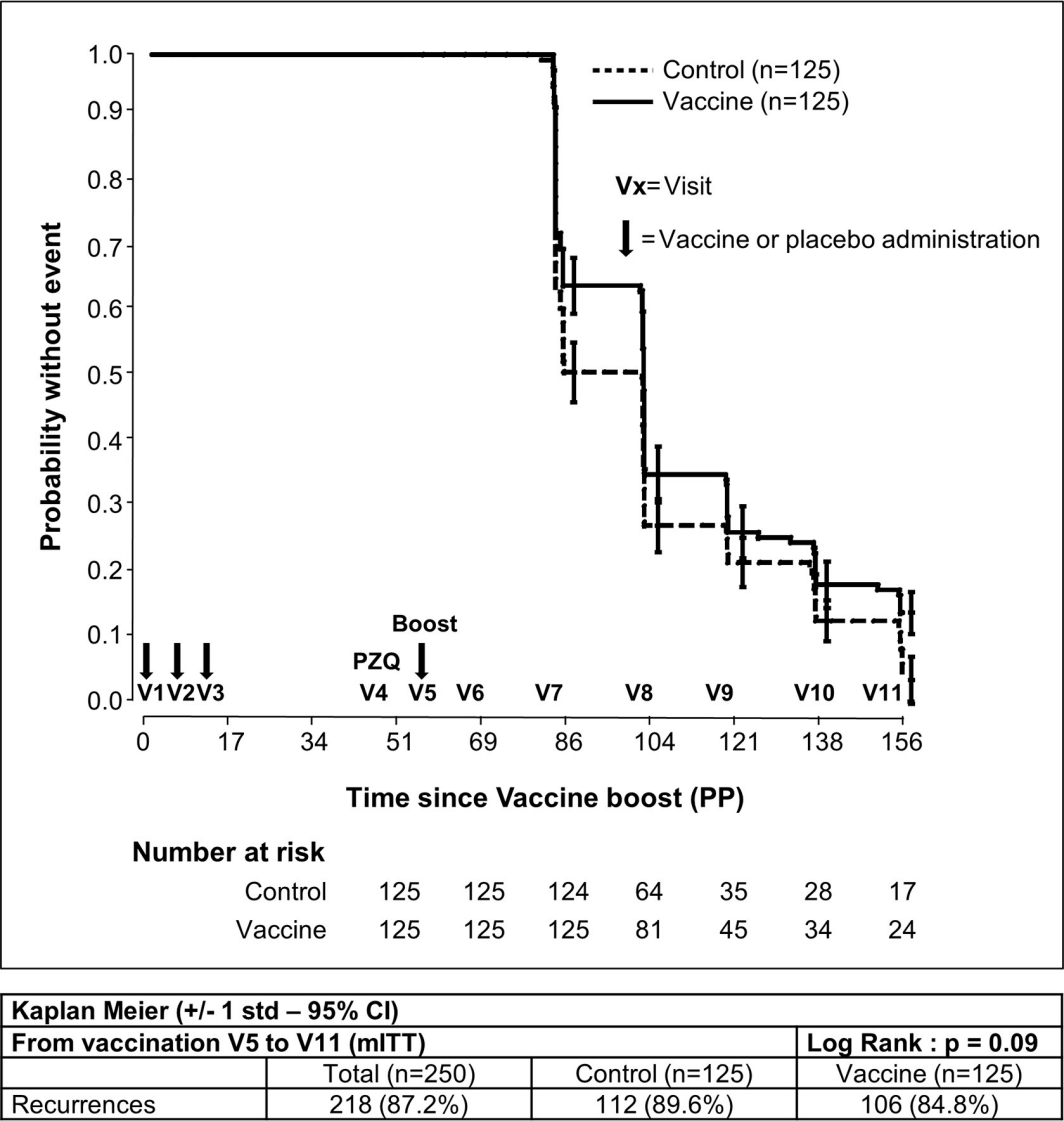
Kaplan–Meier estimates of the cumulative probability of not developing schistosomiasis recurrences among participants in the mITT analysis.

The primary efficacy endpoint (the time to onset of the first schistosomiasis infection recurrence) was assessed in the ITT population, comprising all randomly assigned subjects during the 3-year follow-up period (V1 to V11). The median follow-up time for participants without recurrence was 22.9 months (interquartile range: 18.8–23.0) for the Bilhvax group and 18.8 months (interquartile range: 18.8–23.0 months) for the control group (log-rank p = 0.27). At V11, 86.4% subjects had experienced at least one recurrence in the Bilhvax group and 89.6% in the control group. Kaplan–Meier curves illustrating recurrence-free survival probability in Bilhvax and control groups are shown in Fig 3. In the mITT cohort (Fig 4), from V6 to V11, recurrences of urinary schistosomiasis were documented in 84.8% of the vaccinated individuals and in 89.6% of the control group. No significant difference was observed between the two groups (log-rank p = 0.09). When results were adjusted for sex or age groups (6–7 and 8–9 years) no differences were observed, either. Secondary efficacy endpoints defined as the percentage of subjects without recurrence and the number of recurrences per subject was similar in the two groups (S3 Table).

Vaccine immunization did not affect parasitological manifestations (number of eggs, number of viable eggs, number of hatched eggs, percent of eggs hatching) at the first recurrence or at the end of the trial (V11) between groups (S4 Table) or the number of *Sm* co-infected participants (S5 Table). Children were also examined by ultrasonography to assess *Sh-*related urinary tract morbidity using *Sh* score during visits Vp2, V1, V6, V7, V8, V9, V10, and V11. There were no significant differences between control and Bilhvax groups (S1 Fig).

### Immunogenicity

**Fig 5:**
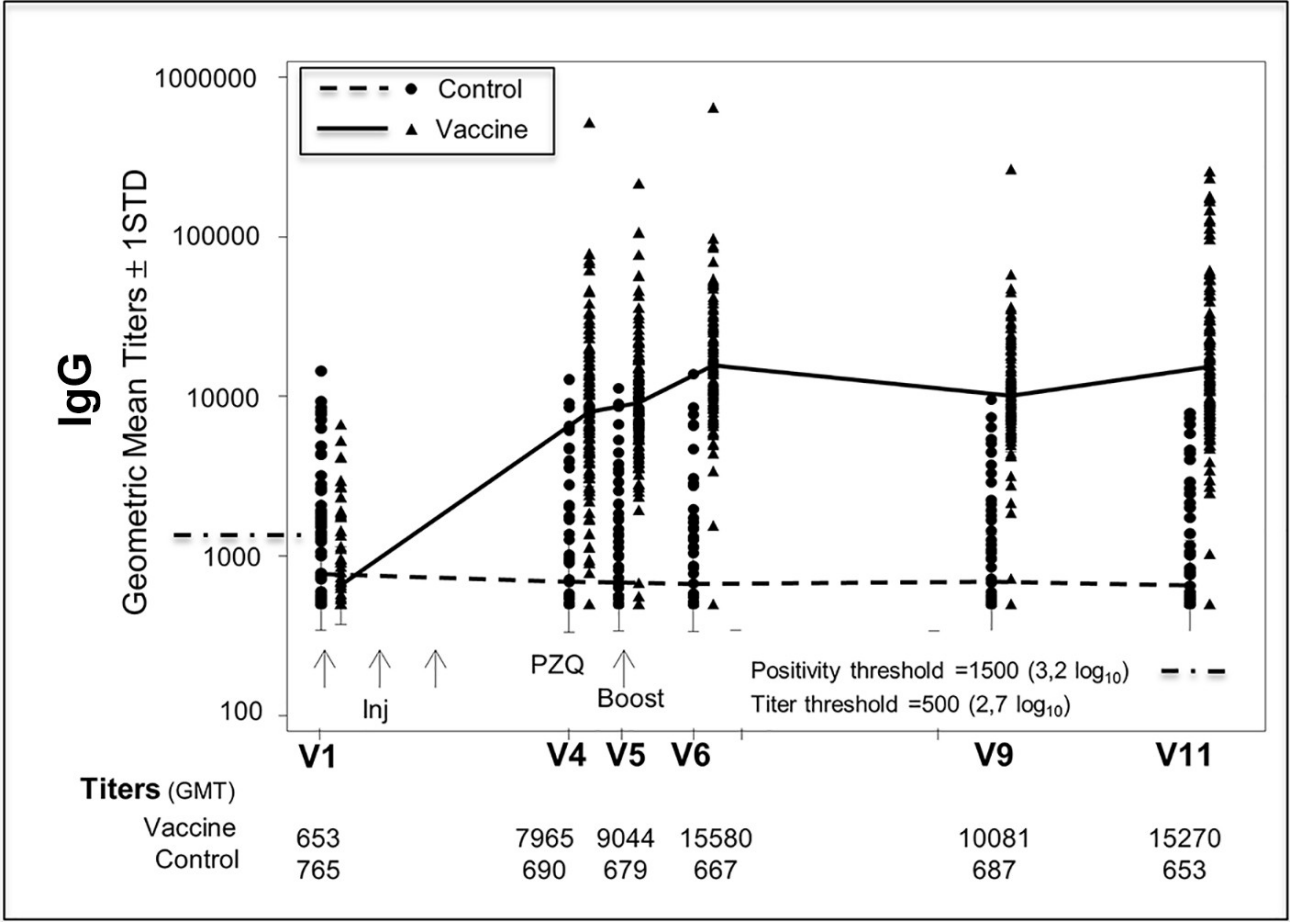
Anti-Sh28GST IgG antibody levels in placebo- and vaccine-treated groups from V1 to V11.

**Fig 6:**
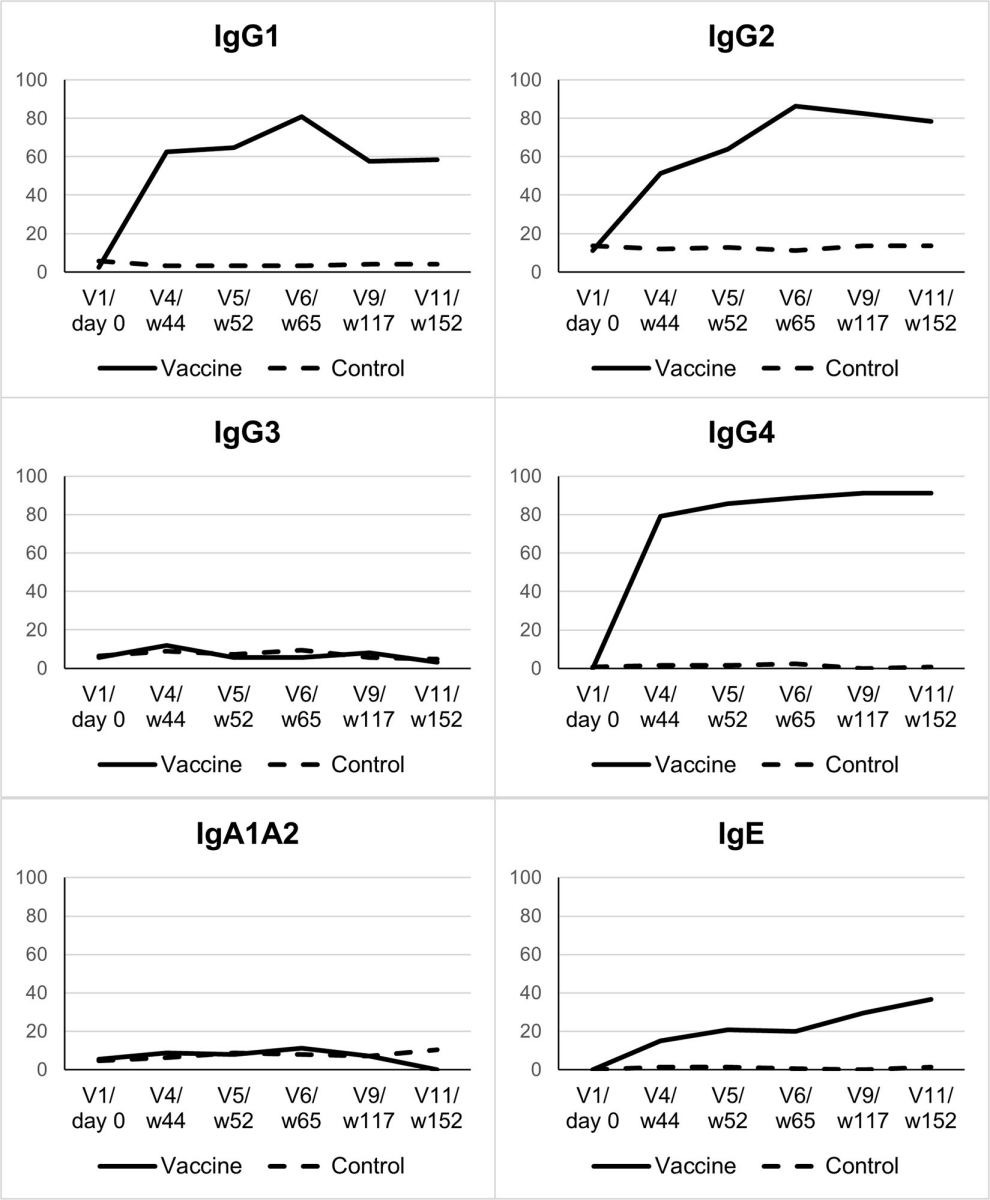
Percentage of responder subjects to the indicated anti-Sh28GST Ig isotypes in placebo- and vaccine-treated groups from V1 to V11. Arrows indicate the time schedule of Sh28GST injections.

Specific antibody response to rSh28GST was measured in Bilhvax and control groups at V1, V4, V5, V6, V9, and V11. Between V1 and V4 (post-vaccination), anti-rSh28GST total IgG geometric mean was significantly increased (p<0.001) in vaccinated children, and the difference between Bilhvax and control groups persisted until the end of the follow-up period (Fig 5). When IgG isotypes were considered, antiSh28GST IgG1, IgG2, and IgG4 antibody titers significantly increased after vaccination in the Bilhvax versus control group (Fig 6). The percent of positive subjects at V11 reached 58.4% (IgG1), 78.4% (IgG2), and 91.2% for IgG4 (Fig 6). The increase in the specific IgE antibody response was more modest, with only 36.8% positive children in the vaccine group at V11. In contrast, no significant difference in anti-rSh28GST IgG3 or IgA antibodies was observed between Bilhvax and control groups from V1 to V11 (Fig 6).

An important aspect of the generated immune response is the inhibition of rSh28GST enzymatic activity by antibodies from vaccinated animals that was previously described to be related to the anti-fecundity effect of 28GST, preventing development of schistosomiasis pathology in experimental models [24]

The capacity of sera to inhibit rSh28GST enzymatic activity is presented in S6 Table. At each point after vaccination, the mean capacity of individual sera samples to inhibit rSh28GST enzymatic activity was significantly higher in Bilhvax compared with the control group (p<0.0001). The percent sera with neutralizing antibodies (≥10% inhibition) reached more than 70% (71.2 to 95.0%) in vaccinated subjects versus 8% maximum in controls. Considering the threshold of 60% inhibition, where any of the sera of the control group reached this level of inhibitory activity, more than 52% of sera samples from the vaccine group did so at one visit (V6). In this group, 82.4% of the subjects (103) reached serum inhibitory activity ≥60% at least once during the post-vaccination follow-up.

Enzymatic inhibition is expressed a percentages. The percentage of inhibition was calculated by the ratio of GST activity after serum incubation to control GST activity. This value is considered significantly positive above 10% (n = 125 for the vaccine group; n = 125 for the control group from V1 to V6 and then 124 at V9 and V11).

## Discussion

To our knowledge, Bilhvax (rSh28GST adjuvanted with Alhydrogel) is the first schistosomiasis candidate vaccine to reach a phase 3 clinical trial, aiming a significant delay in *Sh* infection recurrence in vaccinated children aged 6–9 years compared to a control group in a hyperendemic area of Senegal. The major finding of the study is that during the 3 years of the trial, no significant differences between Bilhvax and control groups were found at the level of urinary schistosomiasis, whatever criteria were considered. However, several results obtained over the course of this specific trial have to be discussed because they could be of interest in the general context of vaccine against schistosomiasis in trial designs in endemic areas.

The potential of the schistosome P28GST to provide protection in prophylactic protocols has been demonstrated in the case of *Sm*, *Sh*, and *S. bovis [1, 10, 25, 26]*. In these preclinical studies, the vaccine significantly inhibited female worm fecundity and egg viability, which indicated its therapeutic properties in schistosomiasis. This result led to Bilhvax phase 1 and 2 clinical trials of Sh28GST, pointing to the conclusion that Bilhvax was safe, both in adults and children, including those with infection. In infected subjects, Bilhvax was highly immunogenic, inducing an elevated Th2 response, and could be administered in combination with one dose of PZQ (*Riveau et al*, *in preparation*). The Bilhvax 3 trial was thus planned according to this combination therapy protocol, presenting the main advantage of being included in the national program of schistosomiasis control in Senegal, and as such, accepted by the authorities and the local population.

The key strengths of this study were acceptability by the population (parents and children), participation of the school teachers, and intensive health care during the trial. The trial was conducted to a high ethical and clinical standard (see Study design and participation). Moreover, the protocol intended for school-age children with a rather long period of surveillance (3 years) included a specific clause that recommended individual PZQ treatments each time a child was found to be infected. A perfect adherence to protocol in regard to administration of the treatment strategy (vaccine+PZQ) and to the schedule of this 3-year trial was obtained. One subject dropped out at V7, but no participant was lost to follow-up during the trial.

A good tolerance to the vaccine and to the induced immune response was observed without related serious AEs. The number of reactions related to the injection site was significantly greater in the vaccine group (73%) than in the placebo group (58%). These AEs were mainly grade 2 and 3 reactions at the injection site, occurring predominantly at the time of the vaccine boost injection, and AEs observed at the injection site disappeared after 48 h.

The primary endpoint of Bilhvax efficacy was a significant delay between both groups in schistosomiasis recurrence defined by a microhematuria ≥1+ (urine stick, 25 to 70 RBC/mm^3^) associated with the presence of at least one *Sh* egg. The requirement of at least one *Sh* egg was included as a criterion to measure the main objective to ensure that hematuria was associated with *Sh* recurrence and not with other etiologies (bacterial/viral infections or trauma). This primary endpoint of efficacy was not achieved, as no statistically significant delay in the recurrence of urinary *Sh* infection was observed in the vaccine group compared with the control group at 36 months after the first vaccination.

The lack of a significant delay in schistosomiasis recurrence between both groups despite the induced immunity in the vaccine group may indicate that this primary endpoint might not be suitable for evaluating the effectiveness of the vaccine. It might have been more appropriate to look for an effect of the vaccination limiting the intensity of the infection during post-vaccination follow-up in comparison with the control group. However, this difference in the intensity of infection after recurrence between the two groups could not be observed. Indeed, a systematic treatment at the advent of a re-infection (hematuria 1+ and 1 living egg) renders impossible to detect an effect of vaccination on the infection intensity over time. We do think that even if the 28GST vaccine efficiency did not fall within the primary endpoint of the trial, it could have been evaluated in following the intensity of infection after recurrence if PZQ treatment had not been performed systematically without endangering children, knowing that medical follow-up was continuous and intense.

It can be considered also the influence of the repeated treatment with PZQ each time a recurrence was detected, as requested in the protocol. This factor has not been considered in previous trials and is of main interest when a trial is performed in subjects having a history of infection. Previous studies demonstrated that the widespread use of PZQ may reduce levels of immunity to urogenital schistosomiasis [27]. Indeed, PZQ treatments after vaccination, i.e., during the elaboration of immune response to Sh28GST, might have interfered with the cytokine response, as recently published by Mutapi et al. [28, 29]. PZQ treatment leads to an increase in pro-inflammatory cytokine responses to antigens from whole *Sh* cercariae and eggs, which both express GST [28]. In addition, PZQ treatment increases the number of subjects producing Sh28GST-specific pro-inflammatory cytokines (TNFα, IL-6, and IL-8) as well as Th1-associated cytokines (IFNγ, IL-2, and IL-12p70) cytokines, as well as Th17-associated cytokine IL-23p19 [29].

The immune response induced was consistent because 99% of the vaccinated individuals were up to the threshold. This specific response was already significantly increased before the booster (V5) and was persistent 2 years after the booster. The anti-Sh28GST response showed elevated levels of specific IgG1, IgG2, and IgG4 (major) antibodies but with an unexpected absence of IgG3 and IgA antibodies. Functionally speaking, a strong capacity of sera from the vaccine arm to inhibit the enzymatic activity of rSh28GST was observed, with a high proportion of vaccinated subject sera (74%) reaching ≥60% of inhibition.

Contrary to what was expected, the administration of PZQ before the booster showed no significant effect on the intensity of the specific response in the vaccine group. When the immune response induced by the vaccine is considered, the absence of specific IgG3 antibodies in the vaccine group, together with the low IgA and IgE levels, might represent a key factor involved in the non-efficacy of the vaccine. Indeed, our previous studies have shown elevated anti-Sh28GST IgG3, IgE, and IgA antibodies compared to IgG1, in association with acquired immunity against reinfection in urinary schistosomiasis [30]. Immunoepidemiological studies in human populations indicate that the presence of IgG3-specific antibodies correlates with naturally acquired protective immunity against schistosomiasis [31]. A phase 1 clinical trial conducted in healthy subjects [12] revealed that rSh28GST induced high IgG3 antibody titers, an effect that was associated previously with reduced egg production and decreased tract urinary pathology in *Sh* infection (23).

In the present clinical trial, the immune response was evaluated after the third injection of the vaccine candidate. The results show the absence of induction of IgG3 antibodies, whereas in contrast, the IgG4 levels were highly increased. In previous clinical trials, IgG3 production was observed following two administrations of the vaccine (phase 1 (12) and phase 2 (in preparation)). It seems therefore likely that, when isotypic response is considered, the third administration induced a rapid isotypic maturation, favoring a production of IgG4 to the detriment of other isotype production. This observed switch may be due to a increase in IL-4 production and action of specific CD4+ T-cells [32]. When drafting the study protocol, it has been hypothesized that induction of high immune titers should provide a better demonstration of the protective effect of the vaccine candidate. As a result, we chose to proceed to a powerful vaccination protocol including three administrations in primo immunization combined with a boost one year after the first injection. Quantitatively, the immune responses obtained have been strong and lasting. However, considering the main criteria, it would be more rational to question the isotypic quality of the specific immune response rather than its amplitude. In addition, it is quite likely that the preexisting antigen response in the included subjects, or at least the existence of immune memory for 28GST, may influence the immune response to the vaccine, notably in terms of isotypic orientation. This could be a decisive argument in favor of reducing the number of vaccine injections (for example in avoiding the boost injection) in a population that has been confronted with the parasite.

In the group of the youngest children (6 and 7 year-old) that we found the most subjects with a pre-existing immune response to the antigen. In this population of 6 to 9 years in which 30% already have an anti-28GST response, youngest children have an acquired anti-28GST response superior to that of 9-year-olds. Considering that this specific immune response can negatively influence the quality of vaccination, it would have been appropriate to target a much younger age group. Nevertheless, whether we cannot say that a pre-existing anti-28GST response has an influence on the final vaccine immune response, no quantitative and qualitative difference in induced immune response was observed between the responders before vaccination and those who were "naive" to the antigen.

An alternative hypothesis is related to the nature of the adjuvant, which was selected for its unique capacity to induce a strong Th2 response and because of its relative safety in large-scale vaccination programs, including in children, all over the world. There is indeed a limited choice of adjuvants presenting similar properties and already in the clinics. Some adjuvants with immunoregulatory properties are currently developed, such as GLA (glucopyranosyl lipid adjuvant; IDRI, Seattle). The use of such an adjuvant may have resulted in a more balanced isotypic response than that observed in the present trial [33], while limiting probably the number of administrations.

Preclinical experiments reported that the vaccine-induced immune response was associated with the detection of antibodies neutralizing the GST enzymatic activity of the vaccine. This observed association in experimental models suggested that inhibition of P28GST enzymatic activity after vaccination was strongly associated with an effect on worm fertility and thus with the protective role of the vaccine [34]. In addition, it has been observed that adult infected people, with an acquired immune response showing an inhibitory activity to 28GST, presented a significantly reduced infection intensity compared to the majority of the studied population (27). This observation in a human population exposed to schistosomiasis corroborates with the numerous results observed in animals during experimental infection. Nevertheless, the inhibition of 28GST by sera from infected individuals is significant but extremely reduced in comparison with the inhibitory capabilities of sera from individuals immunized with the 28GST protein.

In the present study, sera from vaccinated subjects showed a highly significant inhibitory activity indicating the particular affinity of the induced antibodies for the active site of the rSh28GST enzyme. However, the absence of protection despite the strong and persistent inhibitory activity might indicate either that the inhibition of GST activity is unrelated to the protection previously observed in preclinical experiments, or that the criteria for the primary efficacy endpoint were not appropriate, as has been suggested above. It should be noted that the mode of action of schistosome 28GST is not limited to detoxifying oxidized radicals by its GST function as measured by the enzyme assay used in this study. Indeed, 28GST is also a molecule allowing the transport of hormone that may play a role in the reproduction of the worm (7). It is probably that despite the enzymatic activity was inhibited by specific antibodies, this transporter function is not hampered due to the absence of related antibody production. Indeed, the active site of the enzymatic activity is well known, but the one involved in the hormonal transportation is not. It is therefore conceivable that the production of inhibitory antibodies to the enzymatic activity is not related to a protective potential that would be related in fact to the inhibition of hormone binding. This hypothesis remains to be demonstrated.

In contrast with the highly significant protection obtained in vaccine trials performed in non-human primates and in cattle, the results of this first phase 3 trial in humans appear somewhat disappointing. Although the reasons for this failure are likely due to multiple factors, emphasis should be given to the nature of the antibody response and specifically to isotype selection. Immunity to schistosomes is generally regarded as Th2-mediated and antibody-dependent. In human populations, acquisition of immunity is correlated with IgG3 and IgA antibodies to P28GST. The failure to induce the desired isotypic response seems related to three factors: isotypic variation induced by repeated PQZ treatment, use of a non-optimal adjuvant, and repeated vaccine administrations, all favoring production of IgG4 antibodies known to act generally as “blocking” antibodies. IgG4 antibodies occur following chronic exposure to antigen and are generally associated with states of immune tolerance [35]. The control of these factors will be essential for the subsequent trials. It also should be pointed out that this vaccine trial aiming at reducing pathology has been undertaken in highly infected children, who also were thus undergoing chemotherapy.

Two of the positive results of this trial are certainly the safety of the vaccine in children and the duration of the specific immune response. Later trials must thus be reassessed in the absence of PZQ administration, preferably in non-infected younger children, with another pro-Th2 adjuvant.

Regardless, the strong immunogenicity of Sh28GST, its safety of administration, including in children, together with the confirmed anti-worm fecundity effect observed in several models of schistosome infections, make use of Sh28GST as an anti-schistosome vaccine still feasible and encourage further trials.

## Supporting information

S1 Fig Results of ultrasound echotomography. Echotomography score is the sum of vesical, ureteral and renal scores. Vaccined group: solid line; control Group: dotted line.
(TIF)

S1 Table Local or regional adverse effects that may be directly related to treatment. Data are reported as number of children (%) that experimented at least one adverse effect during the study (children may have experimented several adverse effects).
(DOCX)

S2 Table Serious adverse events ≥3. Data are reported as number of events and grade. * A single child presented both malaria episode and gastroenteritis during the course of the study.
(DOCX)

S3 Table Number of recurrences per subject in control and vaccine groups. (DOCX)

S4 Table Parasitological manifestations at time of 1st recurrence (V1 to V11). (DOCX)

S5 Table Number of subjects infected with *S*. *mansoni*. (DOCX)

S6 Table Inhibition of rSh28GST enzymatic activity by sera. (DOCX)
