# Supplementary material for: Safety and efficacy of the rSh28GST urinary schistosomiasis vaccine: A phase 3 randomized, controlled trial in Senegalese children
Source: PLoS Negl Trop Dis. 2018 Dec 7;12(12):e0006968. doi: 10.1371/journal.pntd.0006968 (PMC6300301; doi:10.1371/journal.pntd.0006968)
Supplement: S1 Table — Data are reported as number of children (%) that experimented at least one adverse effect during the study (children may have experimented several adverse effects). (DOCX) [file pntd.0006968.s002.docx]

| **Localization and type of effect** | **Control (n=125)** | **Vaccine (n=125)** | **Total (n=250)** |
| --- | --- | --- | --- |
| ***Effects at injection site***  Pain  Pruritus  induration  swelling | 61 (49%)  0 (0%)  31 (25%)  0 (0%) | 80 (63%)  4 (3%)  70 (56%)  5 (4%) | 141 (56%)  4 (2%)  101 (40%)  5 (2%) |
| ***Regional effects***  Upper arm pain  Upper arm heaviness | 9 (7%)  0 (0%) | 7 (6%)  1 (1%) | 16 (6%)  1 (0.5%) |
