# Supplementary material for: Safety and efficacy of the rSh28GST urinary schistosomiasis vaccine: A phase 3 randomized, controlled trial in Senegalese children
Source: PLoS Negl Trop Dis. 2018 Dec 7;12(12):e0006968. doi: 10.1371/journal.pntd.0006968 (PMC6300301; doi:10.1371/journal.pntd.0006968)
Supplement: S2 Table — Data are reported as number of events and grade. * A single child presented both malaria episode and gastroenteritis during the course of the study. (DOCX) [file pntd.0006968.s003.docx]

| **Type** | **Control (6 / 125)** | **Vaccine (3 / 125 )** |
| --- | --- | --- |
| Abdominal trauma | 1 (grade 3) | 0 |
| Arm fracture | 1 (grade 3) | 0 |
| Gastroenteritis | 0 | 1^*^ (grade 3) |
| Hand phlegmon | 1 (grade 3) | 0 |
| Malaria episode | 0 | 2^*^ (grade 3 and 4) |
| Surgery for inguinal hernia | 2 (grade 3) | 0 |
| Surgery for umbilical hernia | 1 (grade 3) | 0 |
| Anemia (thalassemia trait) | 0 | 1 (grade 3) |
