# Supplementary material for: Safety and efficacy of the rSh28GST urinary schistosomiasis vaccine: A phase 3 randomized, controlled trial in Senegalese children
Source: PLoS Negl Trop Dis. 2018 Dec 7;12(12):e0006968. doi: 10.1371/journal.pntd.0006968 (PMC6300301; doi:10.1371/journal.pntd.0006968)
Supplement: S3 Table — (DOCX) [file pntd.0006968.s004.docx]

|  | **No recurrence** | **1 recurrence** | **2 recurrences** | **3 recurrences** | **4 recurrences** |
| --- | --- | --- | --- | --- | --- |
| **Control** | 13 /125 (10%) | 37/125 (30 %) | 47/125 (38%) | 24/125 (19%) | 4/125 (3%) |
| **Vaccine** | 17 /125 (14%) | 43 /125 (34%) | 48 /125 (38%) | 14 /125 (11%) | 3 /125 (2%) |
