# Supplementary material for: Safety and efficacy of the rSh28GST urinary schistosomiasis vaccine: A phase 3 randomized, controlled trial in Senegalese children
Source: PLoS Negl Trop Dis. 2018 Dec 7;12(12):e0006968. doi: 10.1371/journal.pntd.0006968 (PMC6300301; doi:10.1371/journal.pntd.0006968)
Supplement: S4 Table — (DOCX) [file pntd.0006968.s005.docx]

|  |  | ***Control (n=112)*** | ***Vaccine (n=108)*** |
| --- | --- | --- | --- |
| **Number of viable eggs** | **Min-Max** | 7 to 289 | 6 to 337 |
|  | **Mean (std)** | 130 (60) | 126 (64) |
|  | **Median [IQR]** | 124 [87 ; 176] | 127 [75 ; 163] |
| **Number of  hatched eggs** | **Min-Max** | 3 to 214 | 4 to 288 |
|  | **Mean (std)** | 96 (52) | 89 (55) |
|  | **Median [IQR]** | 92 [58 ; 145] | 87 [46 ; 128] |
| **Egg hatching (%)** | **Min-Max** | 14 to 100 | 21 to 93 |
|  | **Mean (std)** | 72 (18) | 68 (20) |
|  | **Median [IQR]** | 76 [64 ; 85] | 76 [59 ; 83] |
