# Supplementary material for: Safety and efficacy of the rSh28GST urinary schistosomiasis vaccine: A phase 3 randomized, controlled trial in Senegalese children
Source: PLoS Negl Trop Dis. 2018 Dec 7;12(12):e0006968. doi: 10.1371/journal.pntd.0006968 (PMC6300301; doi:10.1371/journal.pntd.0006968)
Supplement: S5 Table — (DOCX) [file pntd.0006968.s006.docx]

| ***S. mansoni* eggs in feces at** | **Control  (n=125)** | **Vaccine (n=125)** | **Total  (n=250)** |
| --- | --- | --- | --- |
| **1^st^ Sh recurrence.** | 55 (49%) | 51 (47%) | 106 / 220 (48%) |
| **V11** | 33 (27%) | 44 (35%) | 77 / 249 (31%) |
