# Supplementary material for: Safety and efficacy of the rSh28GST urinary schistosomiasis vaccine: A phase 3 randomized, controlled trial in Senegalese children
Source: PLoS Negl Trop Dis. 2018 Dec 7;12(12):e0006968. doi: 10.1371/journal.pntd.0006968 (PMC6300301; doi:10.1371/journal.pntd.0006968)
Supplement: S6 Table — (DOCX) [file pntd.0006968.s007.docx]

|  | **% Inhibition** (mean + Std) | |
| --- | --- | --- |
| **Visit** | **Control (%n)** | **Vaccine (%n)** |
| **V1** | 0.0 | 0.0 |
| **V4** | 2.0 ± 3.0 (7.2%) | 34.7 ± 30.5 (72.0%) |
| **V5** | 1.5 ± 2.8 (8.0%) | 39,6 ± 29.7 (76.8%) |
| **V6** | 0.0 | 63.0 ± 26.9 (96.0%) |
| **V9** | 0.9 ± 1.7 (4.8%) | 46.3 ± 25.1 (90.4%) |
| **V11** | 0.3 ± 0.6 (1.6%) | 60.1 ± 28.7 (94.4%) |
